# Predictability of sinusoidally moving stimuli does not improve the accuracy of the accommodative response

**DOI:** 10.1038/s41598-021-94642-2

**Published:** 2021-07-26

**Authors:** Antonio J. Del Águila-Carrasco, Iván Marín-Franch

**Affiliations:** 1 Eye and Vision Research Group, Faculty of Health, University of Plymouth, Plymouth, PL6 8BH UK; 2 Computational Optometry, 18230 Atarfe, Spain

**Keywords:** Optics and photonics, Applied optics, Adaptive optics

## Abstract

Previous research work suggests that predictable target motion such as sinusoidal movement can be anticipated by the visual system, thereby improving the accommodative response. The validity of predictable motion for studying human dynamic accommodation is sometimes put into question. The aim of this work was to assess the effect of anticipation along with learning (and motivation, etc.) and fatigue (and boredom, loss of attention, etc.) on dynamic accommodation experiments from a practical perspective. Specifically, changes in amplitude and temporal phase lag were estimated within and between trials as 9 adult observers were instructed to focus on a stimulus that oscillated sinusoidally towards and away from the eye at specific temporal frequencies. On average, amplitude decreased whereas phase increased within trials. No evidence of anticipation or learning was observed either within or between trials. Fatigue consistently dominated anticipation and learning within the course of each trial. Even if the eye is equipped by a *prediction operator* as it is often assumed, fatigue confounds the results from dynamic accommodation experiments more than anticipation or learning.

## Introduction

**Figure 1:**
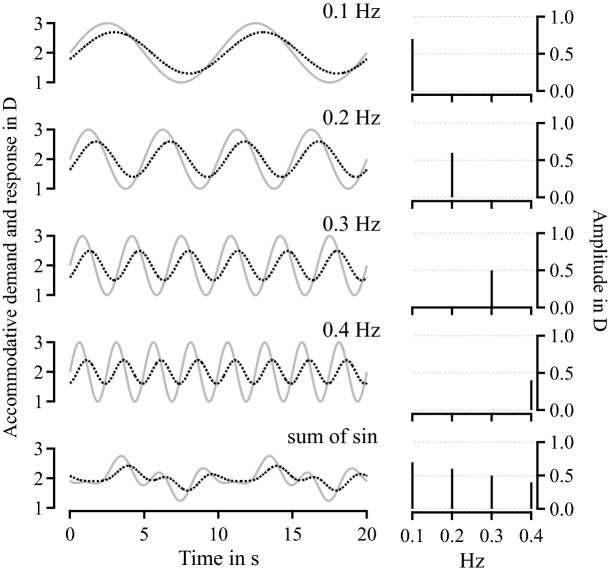
Dynamic accommodation stimulus profiles. The first four rows of the left column show examples of typical and highly predictable sinusoidal accommodative demand profiles (gray curves) at different temporal frequencies and a noiseless accommodative response (solid dotted curve). The last row show an atypical, non-predictable motion profile generated as the sum of the previous four sines and a random phase as those used by Kruger et al.^15^ The graphs on the right column show the amplitude spectra obtained with Fourier analysis of the noiseless response. The amplitude and phase of the accommodative response in these illustrative examples were chosen as they are typical of these types of experiences. For an observer, amplitude tends to decrease and phase tend to increase for faster moving stimuli.

Dynamic accommodation studies typically use stimuli whose dioptric distance from the eye varies sinusoidally after some accommodative cues are removed^1–6^. The impact of any specific optical cues, e.g., binocular disparity, chromatic and monochromatic aberrations, and optical defocus, is assessed by quantifying how well accommodation follows the stimulus. Sine waves are the most used motion profiles in studies of human dynamic accommodation because they are simple to characterize as a function of amplitude—half the distance from crest to trough—in diopters (D) and temporal frequency in Hz. Figure 1 shows four simple sine profiles at temporal frequencies that are commonly used in studies of dynamic accommodation^7,8^ and a complex profile obtained by adding up different sine profiles at random phases^4^.

The underlying response to a sinusoidal movement is typically also a sinusoid with the same frequency but with a smaller amplitude and a small delay or temporal phase lag with respect to the demand^4,5^. For illustration purposes, Fig. 1 also shows noiseless responses to each of the profiles of accommodative demand, ignoring the typical instabilities in optical focus during accommodation known as microfluctuations^9,10^ and other measurement errors due to blinking or eye movements. Amplitude and temporal phase of the sinusoidal response function are inversely correlated since the greater the delay in response, the less the eye needs to accommodate to reach the demand as the stimulus moves in the opposite direction. Therefore, the response amplitude is enough to compare accommodative performance of the eye under different experimental conditions. This basic foundation was used to show that, e.g., chromatic^4^ but not monochromatic^2,3,11^ aberrations are used as monocular cues for accommodation. The same basic foundation was used to show that optical vergence is a stronger cue for accommodation than retinal defocus blur^4,12–14^.

Despite their popularity, the use of sinusoidal changes in accommodative demand for studying human dynamic accommodation has received some criticism due to the repetitive and predictable nature of the visual stimulus^5,6^. Stark et al.^5^ observed shorter time delays in accommodative responses to sinusoidal movements of the target than what they expected for the accommodative system. They concluded that accommodation could be aided by a *prediction operator* when following a repetitive target motion such as a sinusoid. Van der Wildt and colleagues^6^ tested whether anticipation of sinusoidal changes in accommodative demand was important. They reduced the predictability of the sinusoidal temporal profiles by adding noise and observed a systematic decrease in amplitude and increase in temporal phase for the distorted signals. They concluded that observers were able to make use of anticipation when accommodating to the noise-free signal.

As a way to avoid the potential confounding effect of anticipation of the highly predictable sinusoidal target motion, more complex profiles can be designed. Kruger et al.^15^ employed the sum of four sine waves with different temporal frequencies and random temporal phases (as the one shown in the last row of Fig. 1). The problem with the use of these complex signals is that their analysis becomes far more complex. It is necessary to perform a Fourier analysis to compute response amplitude for each of the harmonics that form the accommodative demand profile. For the profile example shown in the last row of Fig. 1 consisting of a sum of four sines, there are four amplitude parameters to analyze. Furthermore, for such complex signals the amplitudes and corresponding phases are no longer inversely correlated, so assessing differences between different experimental conditions may require a multivariate analysis with as many as twice the number of harmonics used to generate the demand profile. Given that Kruger et al.^15^ observed that accommodation was strongest at the fundamental harmonic (0.1 Hz temporal frequency) and contended that observers were not simply responding to predictable rhythmical changes in the target, is the use of complex profiles worth the trouble?

In experimental settings, measurements of this kind are not only susceptible to predictability but also to other confounding effects such as learning, fatigue, and other psychological factors. Anticipation and learning would lead to an improved performance in accommodation over time, whereas fatigue, boredom, and losses in attention would lead to a reduced performance. The repercussion on the measurements is the cumulative effect of all individual confounding factors. If fatigue, boredom, and lack of attention are stronger effects than anticipation and learning, then the amplitude of the accommodative response of observers would decrease over time within trials and between trials, whereas temporal phase would increase. On the other hand, if anticipation and learning effects are stronger, amplitude would increase and phase decrease over time within trials and between trials.

The purpose of this work was to assess the confounding effects of psychological factors such as anticipation, learning, and fatigue on measurements of the accommodative responses to sinusoidal changes in accommodative demand within and between trials. Does learning and anticipation lead to more accurate accommodation, or does fatigue and boredom and lack of attention reduce the accommodative responses? For the sake of simplicity, *fatigue effect* is used here as an umbrella term for all psychophysical and psychological factors (i.e., boredom and loss of attention) that may deteriorate an observer’s performance. Likewise, *learning effect* is an umbrella term for all factors (i.e., motivation and level of attention) that may improve an observer’s performance.

## Methods

### Dynamic accommodation data

The data selected for analysis here was collected during an earlier study of human dynamic accommodation^14^ that examined whether monocular monochromatic accommodation is driven only by a trial-and-error mechanism to minimize blur^16^ or if optical vergence (inverse of target distance) itself is an important cue for accommodation^4,12^. We ensured that all participants showed an amplitude of accommodation greater than 4 D and that they could accommodate to the dynamic monochromatic stimulus. A subset of the conditions employed a visual target that moved sinusoidally at different speeds while a custom adaptive-optics system^13,17^ monitored observer’s accommodative responses and simultaneously corrected their refractive error, astigmatism, and higher-order aberrations. This condition was named the optical-blur condition, since retinal blur was only generated by accommodative inaccuracy, that is, the accommodative response did not match the accommodative demand precisely. The competing condition, for which a blurred target was presented through perfect optics, is not analyzed here. Observers viewed monocularly a monochromatic Maltese cross ($550 \pm 5$ nm) through a 4-mm artificial pupil spanning $1.95^\circ$ of visual angle and with a luminance of about 20 cd m$^{-2}$. The experiments and conditions are explained in full detail in section *2.3 Experimental conditions* and Figure 1 in Marín-Franch et al.^14^.

Data were collected for a total of 9 eyes from 9 observers. The observers had an average (and SD) age of 27(6) years and their refractive errors ranged from $-5.0$ D to $+0.5$ D, with a mean spherical refractive error of $-1.44 (1.89)$ D. None of the observers had astigmatism greater than 1 D. Observers were free from ocular pathologies and accommodation anomalies. The study was approved by the University of Valencia Ethics Committee and informed consent was obtained from each observer. Experiments were performed in accordance with relevant guidelines and regulations and with the Declaration of Helsinki.

The stimulus moved sinusoidally starting at 2 D towards and away from the eye between 1 D and 3 D at temporal frequencies of 0.05 Hz, 0.1 Hz, 0.2 Hz or 0.4 Hz. The initial direction of the target movement was random. The duration of the trials was different for different temporal frequencies (25 s for 0.2 Hz and 0.4 Hz, 30 s for 0.1 Hz, and 40 s for 0.05 Hz). Lower frequency trials had longer duration as to contain at least two cycles of the sinusoidal changes in accommodative demand. For each observer, each experimental condition was repeated 6 times. Therefore, there was a total of 24 trials (6 repetitions at each of the 4 temporal frequencies) per observer. As part of the recruitment process, preliminary trials were run to ensure that observers could accommodate effectively to the monocular, monochromatic target without any optical manipulation, since about 15% to 35% of non-presbyopic adults cannot accommodate under these reduced cue conditions^3,18,19^. These preliminary trials consisted of presentations of a stimulus moving sinusoidally at 0.2 Hz lasting for 25 s. For each observer, the preliminary experiment was repeated 6 times (as it was done with the experimental conditions outlined earlier). Data for these repeat trials were included here also for analysis. Accommodative response was calculated by finding the quadratic surface fit that minimizes the root mean square error of the wavefront, and it was obtained using the Zernike defocus coefficient^20,21^.

Systematic data analysis in Marín-Franch et al.^14^ consisted of fitting a sine wave to the accommodative response, *r*, to a moving stimulus over time *t*, with frequency *f*, amplitude *a*, and temporal phase *p*. Thus,

1 $$\begin{aligned} r = a\, \text {sin}\, 2\pi f \left( t-p\right) , \end{aligned}$$

where the frequency *f* was 0.05 Hz, 0.1 Hz, 0.2 Hz or 0.4 Hz. Amplitude *a* and phase *p* were estimated using ordinary least squares. Note that this calculation is equivalent to performing a Fourier transform and collecting amplitude and phase at the frequency of interest^22^. As we previously published, only three observers could follow the stimulus at 0.4 Hz (Figure 5 in Marin-Franch et al.^14^) and thus, data from this frequency was removed from the analysis. Therefore, data for 24 trials for each of the 9 observers were selected for analysis: 6 preliminary trials and 6 trials for the optical-blur condition with temporal frequencies 0.05 Hz, 0.1 Hz, 0.2 Hz.

### Data analysis

Two analyses were carried out, one quantifying changes in amplitude and phase between trials and another quantifying changes in amplitude and phase over time within single trials. For the first analysis, between-trial data from the preliminary trials was used. Data from the optical-blur condition were not included because the experimental design makes it impossible to obtain reasonable estimates of the changes in amplitude and phase over trials. The reason is that, unlike the preliminary condition that had its 6 repetitions at the same temporal frequency done in succession, for the optical-blur condition trials with slow moving (0.05 Hz and 0.1 Hz) and fast moving (0.2 Hz and 0.4 Hz) stimuli were randomly intermixed among them and also with others from a different experimental condition. Furthermore, because of the large amount of trials, breaks during the experimental session were common, so that observers could rest and follow the stimulus better. For the second analysis, within-trial data from both the preliminary trials and the optical-blur conditions were used as the results are unaffected by trial order and breaks.

For the between-trial analysis, a linear mixed model was used to quantify how amplitude *a*, as estimated with Eq. (1), changed from trial to trial. The model was designed so that there was a common slope quantifying the overall change in amplitude over trials and a different intercept for each of the 9 observers. The Wald method was used to obtain the 95% confidence intervals for the slope representing the overall change in amplitude over trials. The same analysis was replicated for phase *p* as estimated with Eq. (1).

For the within-trial analysis, changes in amplitude and phase were quantified by refitting the accommodative response over time for each trial with a sine wave function for which amplitude and phase was allowed to change over time thus,

2 $$\begin{aligned} r = a(t)\, \text {sin}\, 2\pi f \left( t-p(t)\right) , \end{aligned}$$

where the amplitude and phase functions *a*(*t*) and *p*(*t*) were estimated with a loess-like approach^23^ locally fitting a sine function as in Eq. (1) —instead of a linear or a polynomial function as it is usual. The local-sinusoidal fits were obtained using Gaussian kernels with standard deviations corresponding to a fourth of each trial recording time; that is, 10 s, 7.5 s, and 6.25 s for signals recorded for 40 s, 30 s, and 25 s of accommodative responses to sinusoidally moving stimuli at 0.05 Hz, 0.1 Hz, and 0.2 Hz, respectively. With this method and for each signal, amplitude *a* and phase *p* were estimated at each time *t*. These empirically obtained functions *a*(*t*) and *p*(*t*) were rendered by iteratively moving *t* by 0.1 s and estimating *a* and *p* in each step.

Each amplitude and phase function over time was then fitted a simple linear regression to extract the overall within-trial rate of change in amplitude and phase. A positive slope in amplitude or negative slope in phase would be consistent with anticipation and learning effects, whereas a negative slope in amplitude and positive slope in phase would be consistent with a fatigue effect.

**Figure 2:**
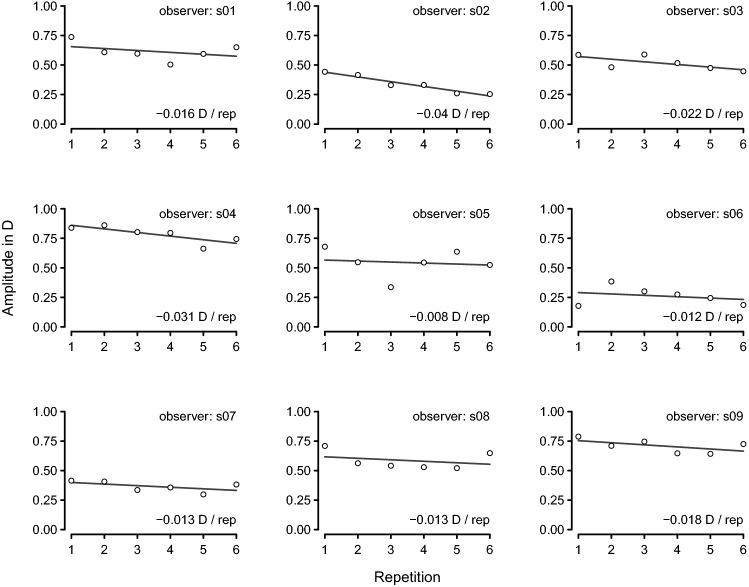
Between-trial amplitude of the accommodative response for each of the 9 observers. The open black circles show the overall amplitude for each of the 6 trials obtained by the least-square fit of the sine function specified in Eq. (1). The black line was estimated by simple linear regression.

There is no reason to think that anticipation, learning, and fatigue effects have to be consistent over time so that the amplitude and phase functions either increase or decrease monotonically. Nevertheless, simple linear regression can only capture the overall change due to the dominating effect over the whole trial. To assess whether the effects of anticipation and learning, and fatigue changed over time, segmented linear regression fits with a single break point (commonly known as a broken-stick or hockey-stick model) were obtained for all trials.

All analyses were performed in MATLAB (Mathworks, Inc., Natic, MA, USA) and the open-source statistical environment R^24^. The R package *lme4*^25^ was used to obtain the estimates from the linear mixed model and corresponding 95% confidence intervals for the between-trials analysis. The package *segmented*^26^ was used to obtain the segmented linear regression for the within-trial analysis. The package provides maximum-likelihood estimates of intercepts and slopes for each segment and of the break point^26^.

## Results

Figure 2 shows the results of simple linear regression for each observer for the between-trial analysis. The estimated changes of amplitude in D per trial for each observer where always negative, ranging from $-0.04$ D to $-0.01$ D per trial. Table 1 shows the results of the linear mixed model fits for the between-trial analysis. According to the fitted models, there was a mild yet clear decrease in amplitude and an increase in phase on the preliminary trials, both significantly different from zero at a significance level of 0.05 as can be deduced from the confidence intervals of Table 1.

**Table 1:** Results of the linear mixed model fits of change in amplitude of accommodative response with repetition number for the preliminary trials.

	Slope	SE	95% CI
Amplitude	0.019	0.005	(0.030, 0.009)
Phase	0.016	0.007	(+ 0.002, + 0.030)

Slope estimates are shown along with their corresponding standard errors (SE) and the 95% confidence intervals (CI). The units of the slopes are D per repetition for amplitude and s per repetition for phase.

**Figure 3:**
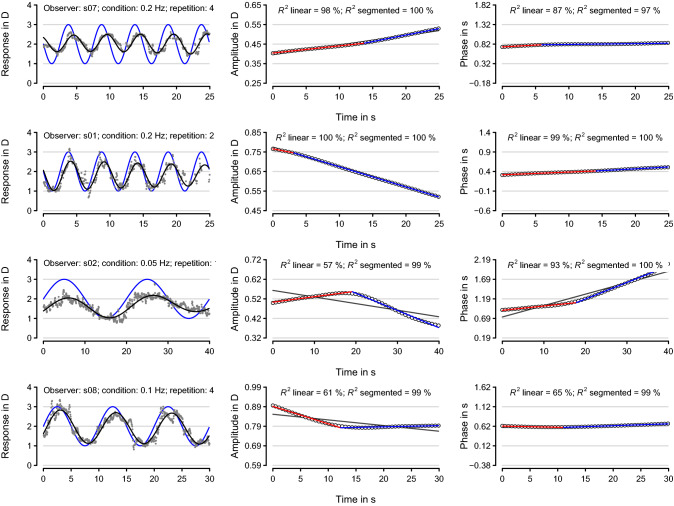
Sample trials for the different experimental conditions. Each row shows a single trial. The left column shows the recorded accommodative response (dark gray dots) to a sinusoidally moving stimuli (blue curve) and the local-sinusoidal fit (black curve) specified by Eq. (2). The observer, condition, and repetition number are specified on the top of each graph. The middle column shows the amplitude in D of the fit in the left column (open black circles) sampled at every 0.5 s. The right column shows the same as the middle column but for phase in s. Simple linear regression fits of amplitude and phase over time in the middle and right columns are shown in dark gray. First and second segments fitted using a broken-stick model are shown in red and blue, respectively. The dark gray line in some of the fits are not visible because they are masked by the red and blue lines fitted by the broken-stick model.

Figure 3 shows four examples (one in each row) of individual response signals along with the corresponding simple and segmented regression analyses for both amplitude and phase over time. The first example corresponds to a trial for which amplitude increased over the whole run, as would be expected if anticipation and learning effects are predominant over fatigue effects. The second example corresponds to a trial for which the amplitude decreased over the whole run, as would be expected if fatigue effects dominate over anticipation and learning effects. The third example correspond to a trial displaying anticipation and learning effect up to a time after which fatigue takes over. The fourth example corresponds to a trial displaying fatigue effect over the first half of the trial after which performance is roughly constant. For the first two examples, the rate of change in amplitude and phase over time are approximately constant and, hence, the lines fitted by the broken-stick model coincide with the simple linear regression fit. Likewise, the broken-stick fit is roughly the same as the linear fit for phase in the fourth example (graph at the bottom-left panel). The supplemental material contains all trials and all conditions along with the calculated amplitude and phase over time for a good accommodator, an average accommodator, and a poor accommodator.

The local-sinusoidal fits (black curves in Fig. 3) were reliable, with only 3 showing clear discontinuities in the amplitude and phase functions as assessed from visual inspection of all 216 fits corresponding to the 24 trials for each of the 9 observers. The 3 discontinuities occurred for two observers and for the same optical-blur condition for a moving stimulus at 0.2 Hz of temporal frequency. Changes in amplitude and phase were often non-monotonic, with only about half of the fits with simple linear regression explaining more than 85% of variance in amplitude (114 out of 216) and in phase (110 out of 216). On the other hand, fits from segmented regression with a single break point explained more than 85% of the variance in more than 95% of fits (207 out of 216 for both amplitude and phase).

**Figure 4:**
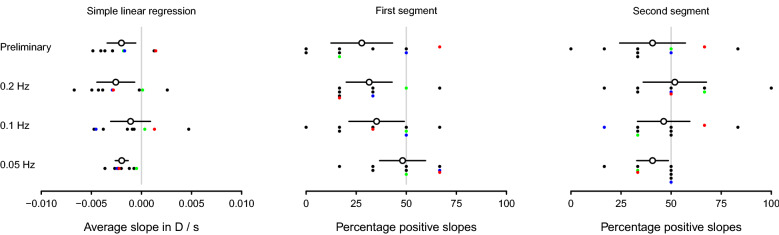
Linear and segmented regression analyses of amplitude. The open circles in the left panel show the average slope estimates in amplitude of accommodation over trials of the preliminary condition and of the 3 optical-blur conditions for moving stimuli at 0.05 Hz, 0.1 Hz, and 0.2 Hz from simple linear regression. The individual slope estimates for each observer are represented as solid black circles, except for the three observers in the supplemental material showcasing a good accommodator, an average accommodator, and a poor accommodator which are represented by solid green, blue, and red circles respectively. The open circles in the middle and right panels show the average percentage of times the slope was positive for, respectively, the first and second regression segments. The individual percentages for each observer are represented as solid black circles, except for the good, average, and poor accommodators in the supplemental material, which are represented by solid green, blue, and red circles respectively. All solid black lines represent the 95% confidence intervals, each with lengths 2 times 1.96 times the standard error of the mean over all 9 observers for each condition.

**Figure 5:**
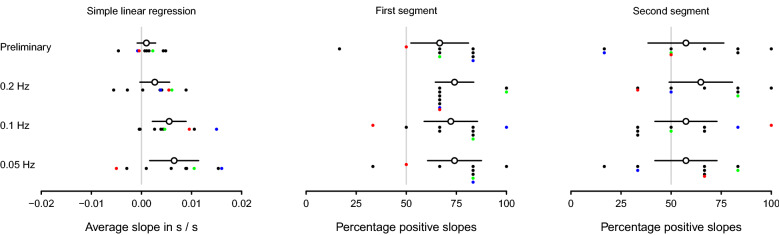
Linear and segmented regression analyses of phase. Details as for Fig. 4 but for slope estimates for phase, instead of amplitude.

Figure 4 shows, for each condition, the average rate of change in amplitude over observers obtained with simple linear regression and the average over observers of the proportion of times the slopes for each segment of the regression were positive from all 6 repetitions for each condition. Figure 5 shows the same as Fig. 4 but for phase instead of amplitude.

For all conditions, amplitude decreased with time on average over the course of each trial (left panel of Fig. 4) and that reduction occurred for most observers in both at start (middle panel of Fig. 4) and end (right panel of Fig. 4) of the trial, with the only exception of the 0.2 Hz optical-blur condition, for which the percentage of positive slopes in the second segment was slightly greater than 50%. Likewise, phase increased with time on average over the course of each trial (left panel of Fig. 5) and that increment occurred for most observers in both at the start (middle panel of Fig. 5) and at the end (middle panel of Fig. 5) of the trial.

## Discussion

Anticipation, learning, motivation, fatigue, boredom, and loss of attention are all confounding effects that may affect accommodative responses during extended and repetitive testing. Anticipation and learning have been argued to be especially important when dealing with predictable sinusoidal accommodative demands^5,6^. However, in the present study, little direct evidence was found of improved responses due to anticipation and learning. On the contrary, declines in amplitude and increases in phase were observed most often. Linear regression for both amplitude and phase showed that fatigue dominated over any potential anticipation and learning effects over the course of each trial and across repeated trials. The observed fatigue effects were generally small with an overall decrease in amplitude of 0.02 D and increase in phase of 0.02 s per trial (Table 1) and from 0.01 D to 0.03 D (Fig. 4) and from 0.01 s to 0.07 s (Fig. 5) for every 10 s of trial run.

These results do not support the conclusions from Stark and colleagues^5^ or those from Van der Wildt et al.^6^. Stark et al. concluded that *when the [crystalline] lens follows simple target motions such as a single sinusoid it evidently is aided by a prediction operator*. This conclusion was based on the observation that latency (time of inactivity before the eye reacts) to step changes in accommodative demand was considerably greater than the temporal phase of sinusoidal changes. Such an inference is speculative, and fails to acknowledge the obvious differences between stimuli: one that abruptly generates a lot of blur (depending on step size) versus one that does so gradually. In addition, the number of observers is unclear (seemingly two, “D. D.” and “J. R.”), yet there were large fluctuations in the decrease of temporal phase^6^.

The lack of power in Stark et al. study^5^ led Van der Wildt and colleagues^6^ to design an experiment to corroborate their conclusion. In order to check for the possibility of anticipation in the response, they removed the predictability of the input signal by masking the sinusoidal signal with enough noise for the observers to recognize the signal. More precisely, they added Gaussian noise with a low-pass filter to up to 5 Hz. They concluded that the systematic decrease in amplitude and increase in phase was due to observers inability to anticipate the noisy signal. But the decrease in amplitude and increase in phase could also be a consequence of the greater stress imposed on the ciliary muscle by demanding it to follow a stimulus that moved at times at much greater speeds (of up to 5 Hz). As calculated with the linear mixed model which estimates are presented in Table 1, amplitude clearly decreases and phase increases with temporal frequency of the accommodative demand. And as shown in Figure 5 of Marín-Franch et al.,^14^ for a temporal frequency of 0.4 Hz, most observers struggle to even follow the moving stimulus.

The approach taken here was far more practical and did not require masking the signal. Even if the accommodation control system really is *aided by a prediction operator* as suggested by Stark et al.,^5^ the results of this analysis point towards a fatigue effect dominating observers responses in these kinds of experiments.

There is evidence for fatigue being an important confounding factor for accommodation in other studies of accommodations. For instance, Lancaster and Williams^27^ observed a decrease of power to accommodate after about half an hour of continuous near fixation. This observation was corroborated by another set of experiments by Berens and Sells^28^, who also studied fatigue of accommodation binocularly and monocularly, while the contralateral eye was patched, and found that both eyes manifested fatigue, even the occluded one. In yet another series of experiments, it was demonstrated that tonic accommodation is reduced after performing a repetitive accommodative task using a lens flipper due to accommodative fatigue^29^. Sharmin and Vohnsen found that fatigue increased the accommodation response time to step-changes in demand by up to three times^30^. There is also evidence against fatigue effects. One study observed that the amplitude of the accommodation did not seem to change systematically during 30 minutes of repetitive accommodation tasks following step changes^31^, but responses were highly variable and only two observers were tested. The data analyzed here correspond to a set of experiments that were not designed to quantify the effects of training on accommodation. Explicit training has been shown to improve accommodation^32–34^. Unlike for experiments exploring the effects of weeks long training^34^, the data analyzed here correspond to naïve observers and all trials for each observer were done on the same day. Therefore, the results of both studies cannot be compared to each other.

The results of this work are based on the use of the Zernike defocus term as a proxy for accommodative response. Other methods could have been used to calculate the accommodative response instead. One such method is the paraxial curvature matching method^21^, which is based on paraxial optics and what has become known as Seidel refraction. Although Del Águila-Carrasco et al.^35^ found a greater amplitude of accommodation to a sinusoidal moving stimulus using Seidel refraction, the trend among observers and conclusions drawn from both analyses were essentially the same. Likewise, a similar conclusion would be expected here had the Seidel refraction been used instead of the Zernike defocus term.

The analysis reported here differed from that of Marín-Franch et al.^14^ in that amplitude and temporal phase was calculated over time in each trial, using a loess-like approach locally fitting a sine function. Nevertheless, the results obtained with this new and more sophisticated analysis are consistent with the ones obtained with the original analysis. The mean difference across observers between the two analyses when the stimulus moved following a sinusoidal with frequencies of 0.05 Hz and 0.1 Hz was lower than 0.02 D (with a maximum difference 0.03 D); and for a frequency of 0.2 Hz, the mean difference was lower than 0.01 D (with a maximum difference 0.02 D). These differences are very small and do not change the significance and conclusions of the original, simpler analysis.

Contrary to what previous work suggested^5,6^, accommodative responses to repetitive sinusoidal changes in accommodative demand are dominated by fatigue confounding effects rather than by anticipation and learning. If anticipation to predictable changes in accommodative demand exists, it is outweighed by fatigue effects. This supports the notion that complex experimental designs aimed at avoiding learning effects may only unnecessarily add burden to observers.

## Supplementary Information

Supplementary Information.

## Data Availability

The datasets analysed during the current study are available from the corresponding author on reasonable request.
