# Supplementary material for: Predictability of sinusoidally moving stimuli does not improve the accuracy of the accommodative response
Source: Sci Rep. 2021 Jul 26;11:15195. doi: 10.1038/s41598-021-94642-2 (PMC8313575; doi:10.1038/s41598-021-94642-2)

Observer 4 (good accommodator): Preliminary trials at 0.2 Hz

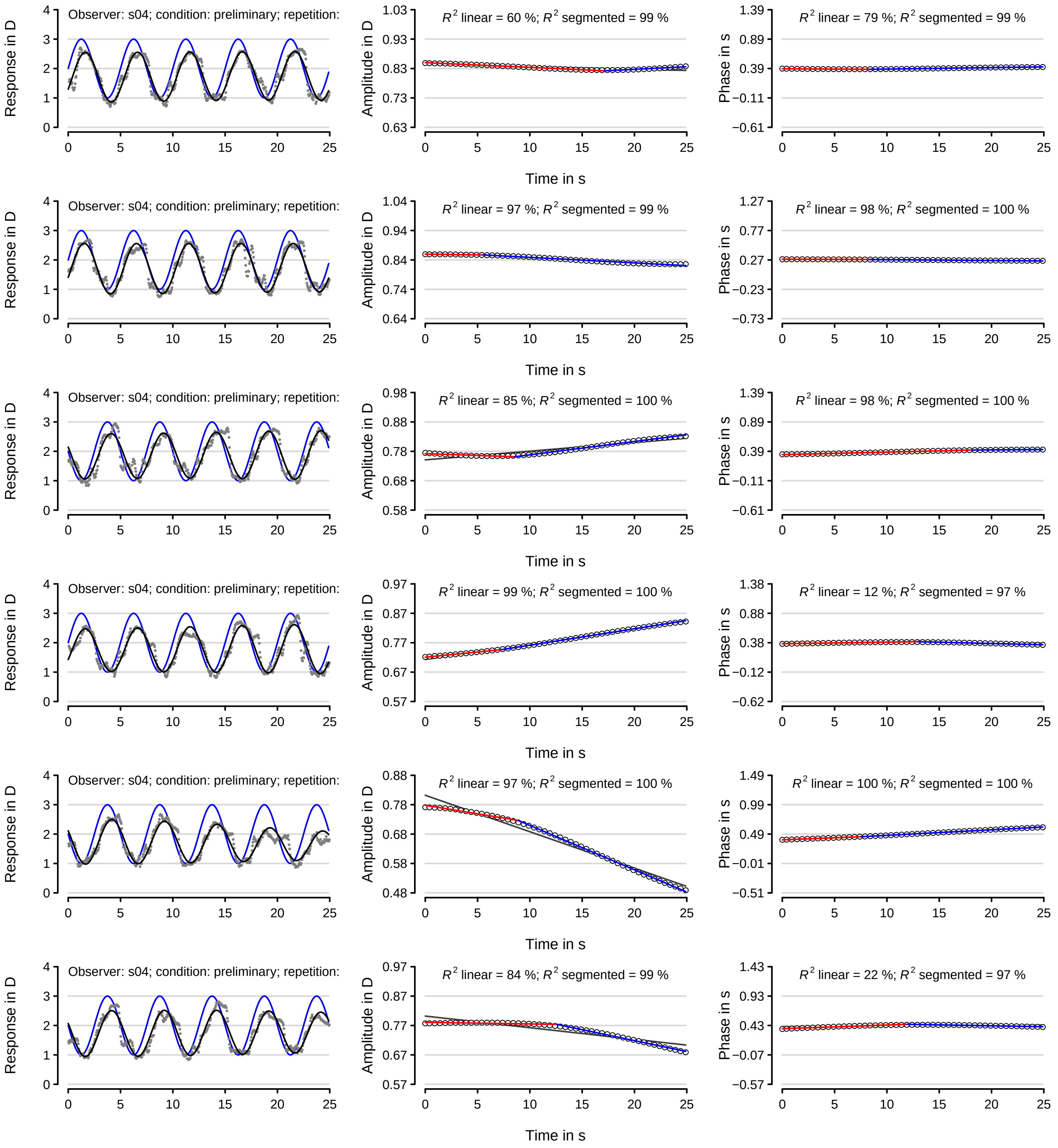

Observer 4 (good accommodator): Optical−blur 0.05 Hz

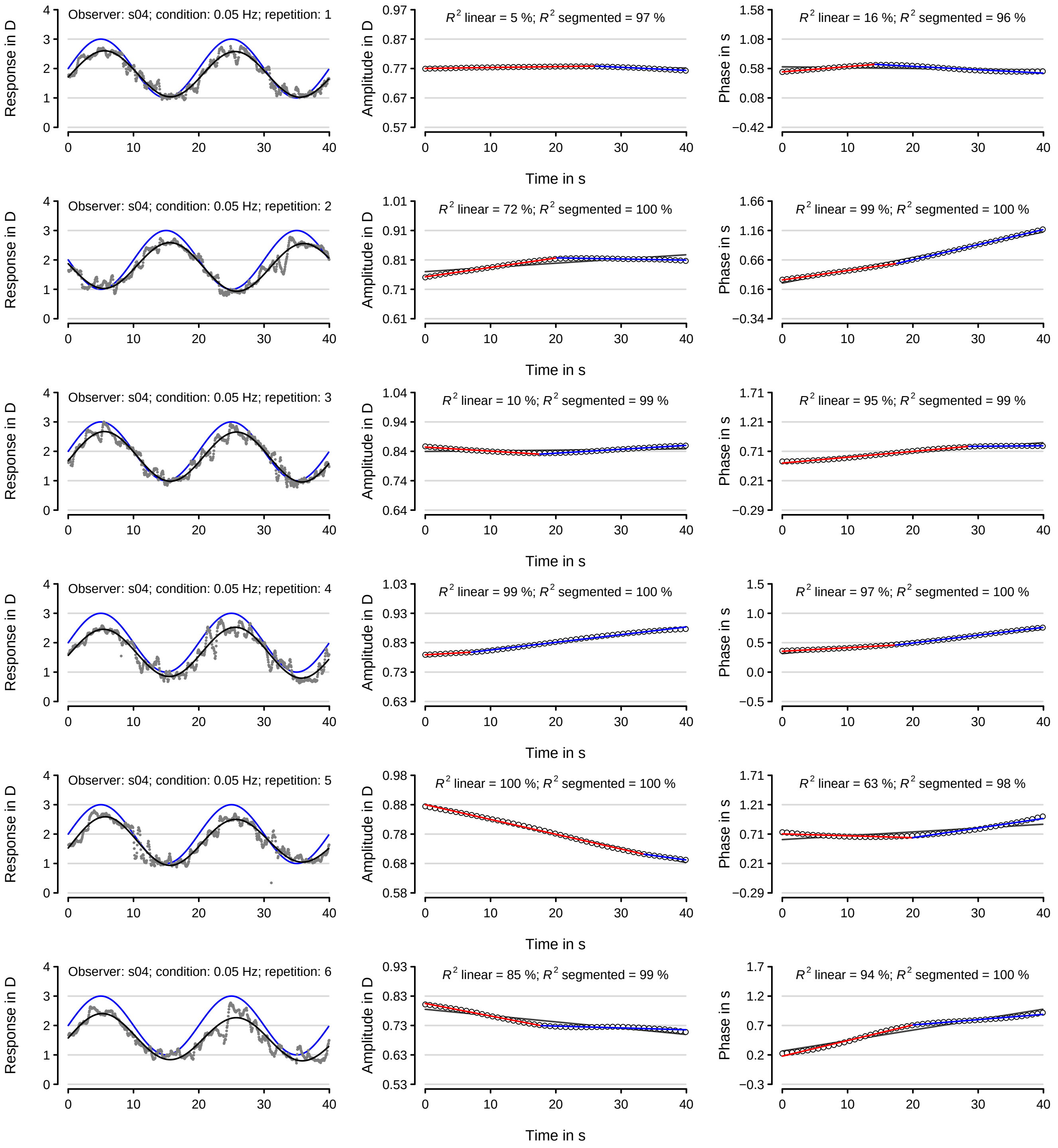

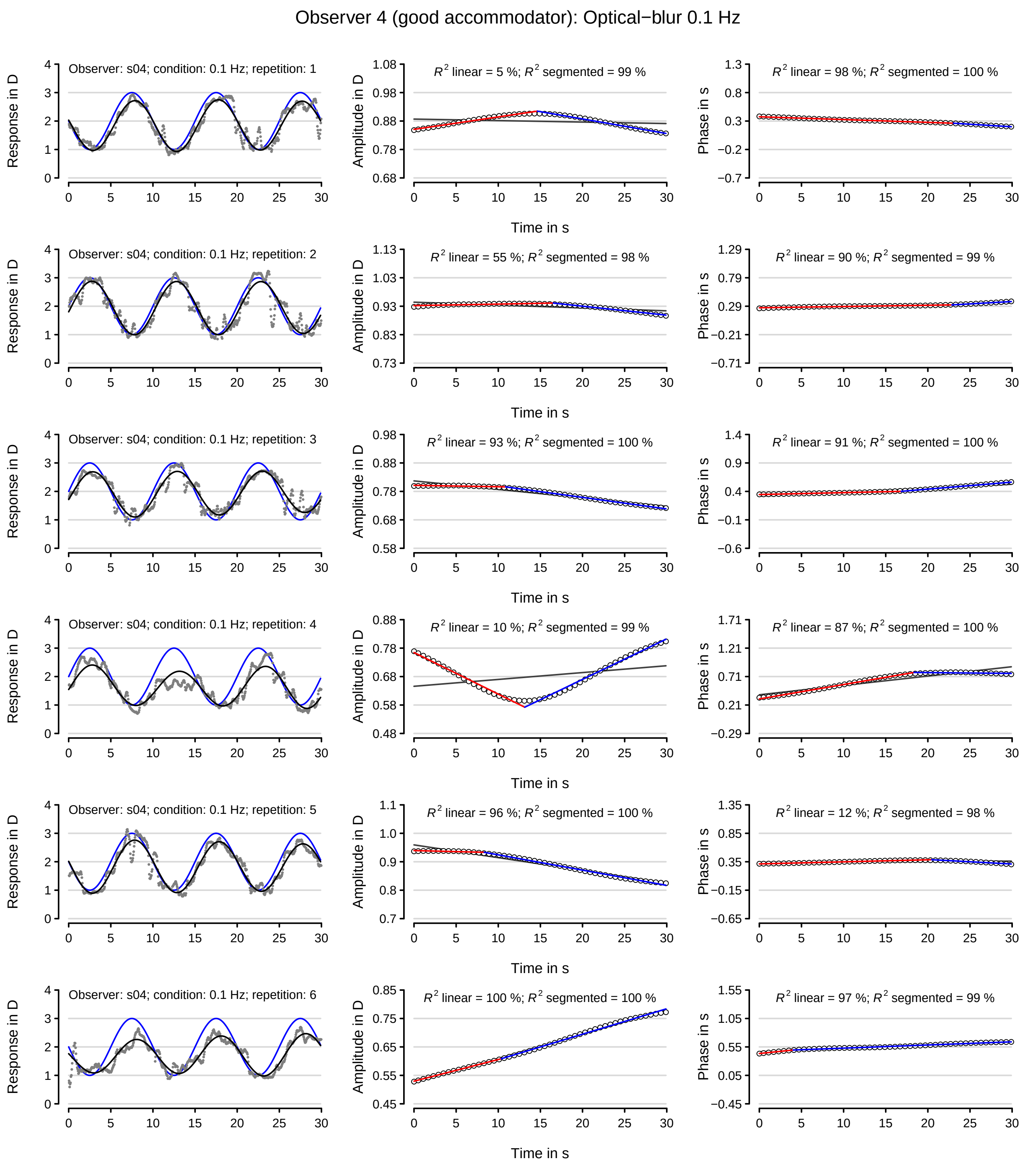
Observer 4 (good accommodator): Optical−blur 0.1 Hz
Observer: s04; condition: 0.1 Hz; repetition: 1
R^2 linear = 5 %; R^2 segmented = 99 %
R^2 linear = 98 %; R^2 segmented = 100 %
Observer: s04; condition: 0.1 Hz; repetition: 2
R^2 linear = 55 %; R^2 segmented = 98 %
R^2 linear = 90 %; R^2 segmented = 99 %
Observer: s04; condition: 0.1 Hz; repetition: 3
R^2 linear = 93 %; R^2 segmented = 100 %
R^2 linear = 91 %; R^2 segmented = 100 %
Observer: s04; condition: 0.1 Hz; repetition: 4
R^2 linear = 10 %; R^2 segmented = 99 %
R^2 linear = 87 %; R^2 segmented = 100 %
Observer: s04; condition: 0.1 Hz; repetition: 5
R^2 linear = 96 %; R^2 segmented = 100 %
R^2 linear = 12 %; R^2 segmented = 98 %
Observer: s04; condition: 0.1 Hz; repetition: 6
R^2 linear = 100 %; R^2 segmented = 100 %
R^2 linear = 97 %; R^2 segmented = 99 %
Response in D
Amplitude in D
Phase in s
Time in s

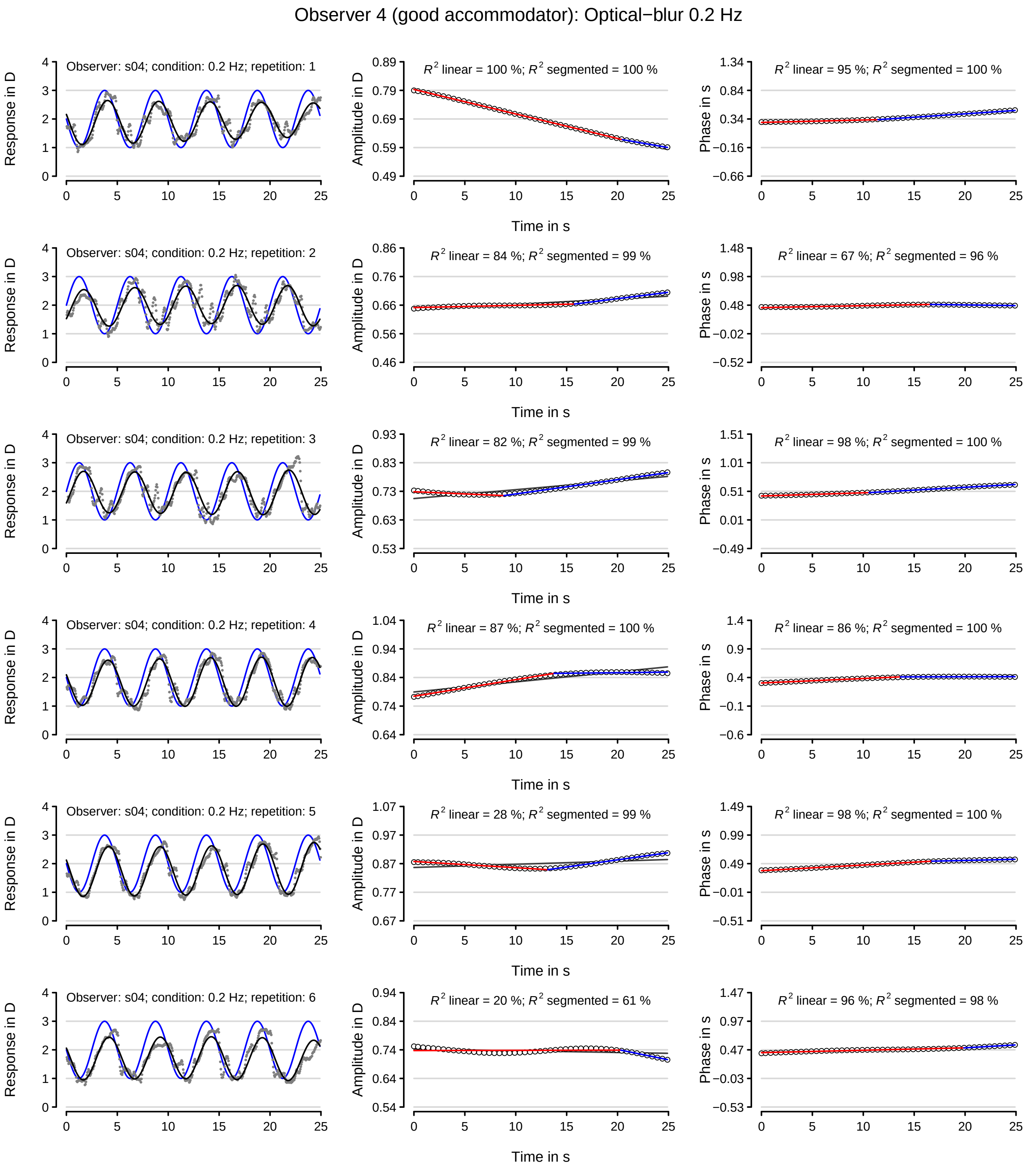
Observer 4 (good accommodator): Optical−blur 0.2 Hz
Observer: s04; condition: 0.2 Hz; repetition: 1
R2 linear = 100 %; R2 segmented = 100 %
R2 linear = 95 %; R2 segmented = 100 %
Observer: s04; condition: 0.2 Hz; repetition: 2
R2 linear = 84 %; R2 segmented = 99 %
R2 linear = 67 %; R2 segmented = 96 %
Observer: s04; condition: 0.2 Hz; repetition: 3
R2 linear = 82 %; R2 segmented = 99 %
R2 linear = 98 %; R2 segmented = 100 %
Observer: s04; condition: 0.2 Hz; repetition: 4
R2 linear = 87 %; R2 segmented = 100 %
R2 linear = 86 %; R2 segmented = 100 %
Observer: s04; condition: 0.2 Hz; repetition: 5
R2 linear = 28 %; R2 segmented = 99 %
R2 linear = 98 %; R2 segmented = 100 %
Observer: s04; condition: 0.2 Hz; repetition: 6
R2 linear = 20 %; R2 segmented = 61 %
R2 linear = 96 %; R2 segmented = 98 %
Response in D
Amplitude in D
Phase in s
Time in s

Observer 3 (average accommodator): Preliminary trials at 0.2 Hz

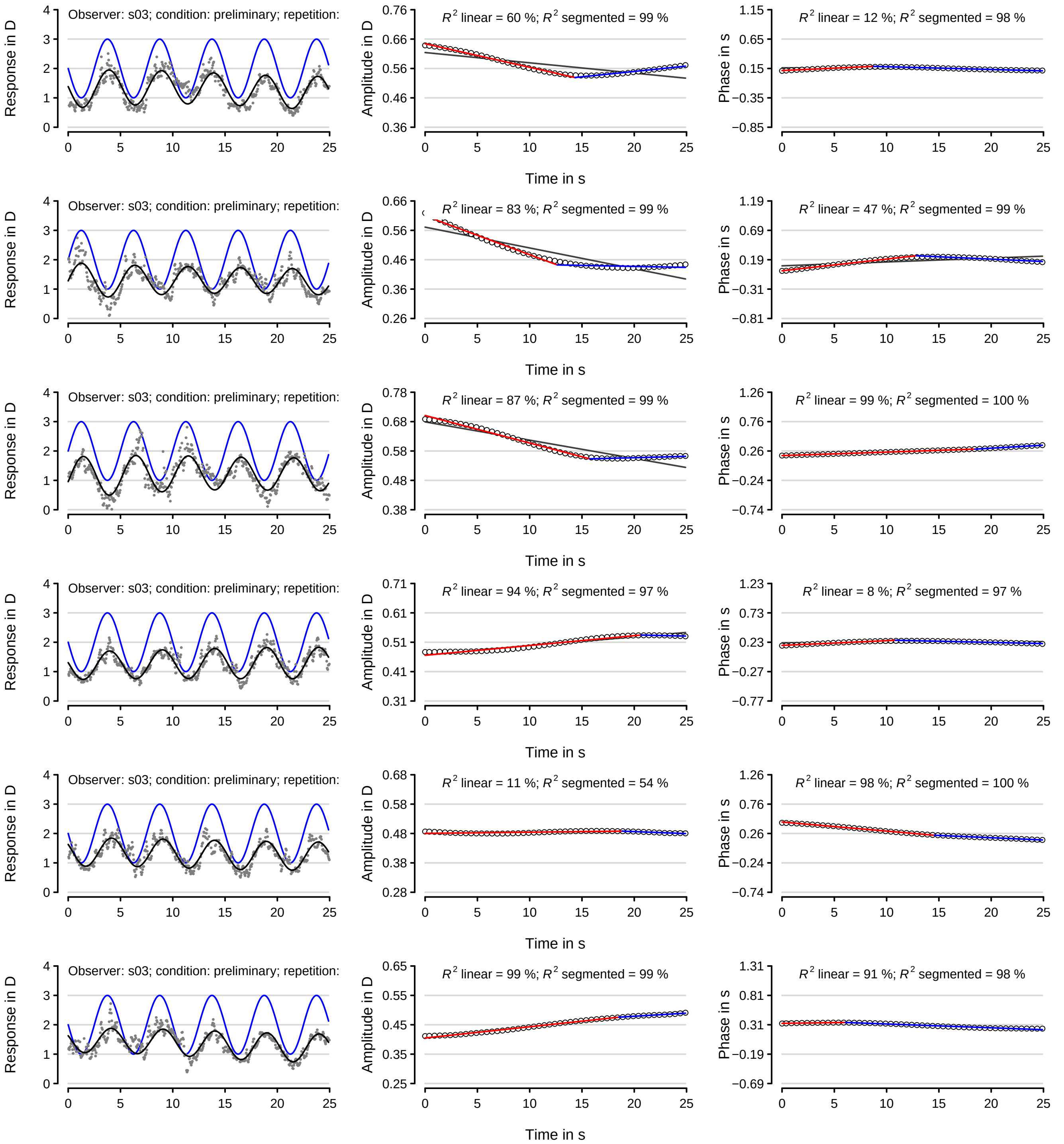

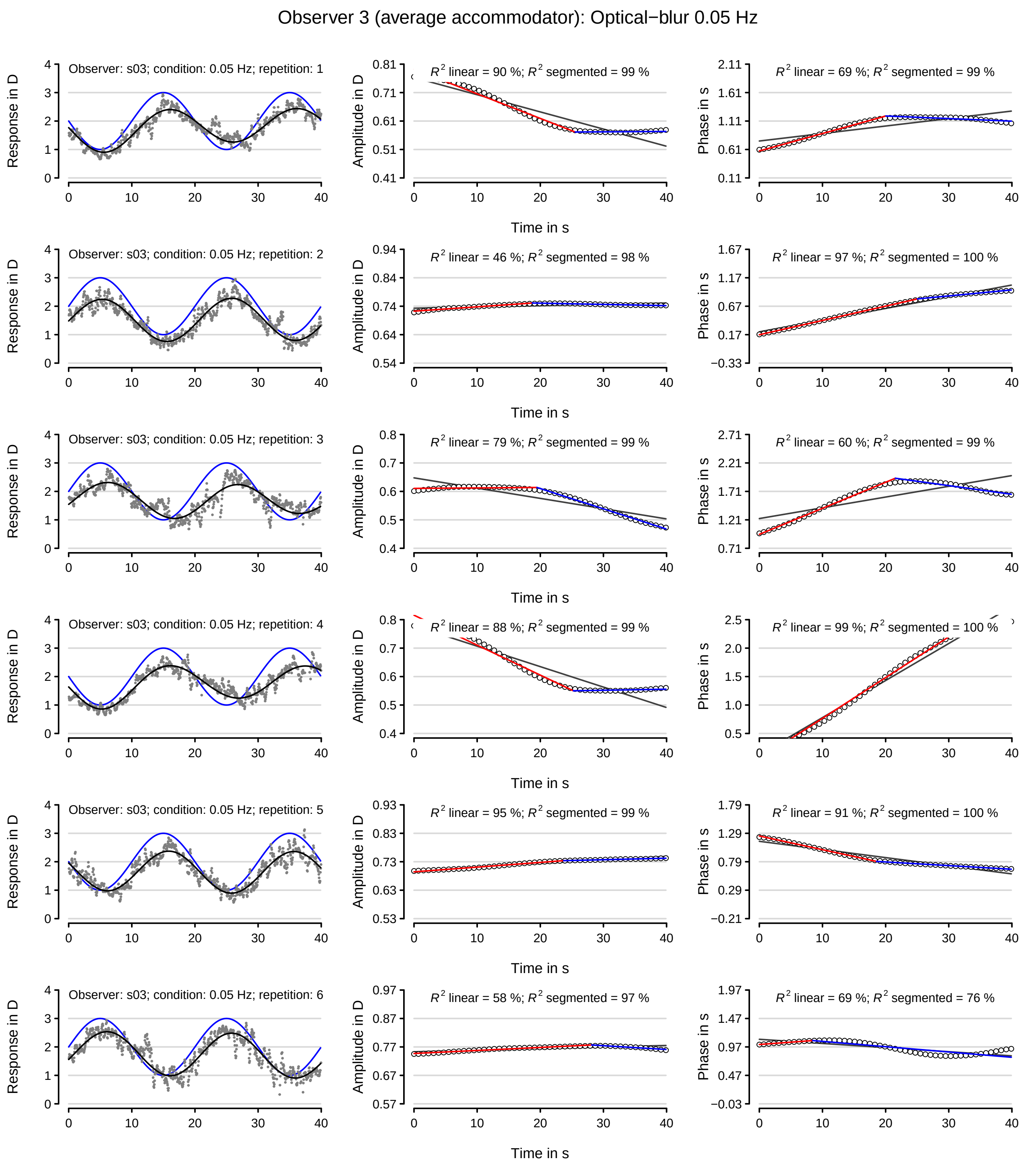

Observer 3 (average accommodator): Optical−blur 0.05 Hz
Observer: s03; condition: 0.05 Hz; repetition: 1
R^2 linear = 90 %; R^2 segmented = 99 %
R^2 linear = 69 %; R^2 segmented = 99 %
Observer: s03; condition: 0.05 Hz; repetition: 2
R^2 linear = 46 %; R^2 segmented = 98 %
R^2 linear = 97 %; R^2 segmented = 100 %
Observer: s03; condition: 0.05 Hz; repetition: 3
R^2 linear = 79 %; R^2 segmented = 99 %
R^2 linear = 60 %; R^2 segmented = 99 %
Observer: s03; condition: 0.05 Hz; repetition: 4
R^2 linear = 88 %; R^2 segmented = 99 %
R^2 linear = 99 %; R^2 segmented = 100 %
Observer: s03; condition: 0.05 Hz; repetition: 5
R^2 linear = 95 %; R^2 segmented = 99 %
R^2 linear = 91 %; R^2 segmented = 100 %
Observer: s03; condition: 0.05 Hz; repetition: 6
R^2 linear = 58 %; R^2 segmented = 97 %
R^2 linear = 69 %; R^2 segmented = 76 %
Response in D
Amplitude in D
Phase in s
Time in s

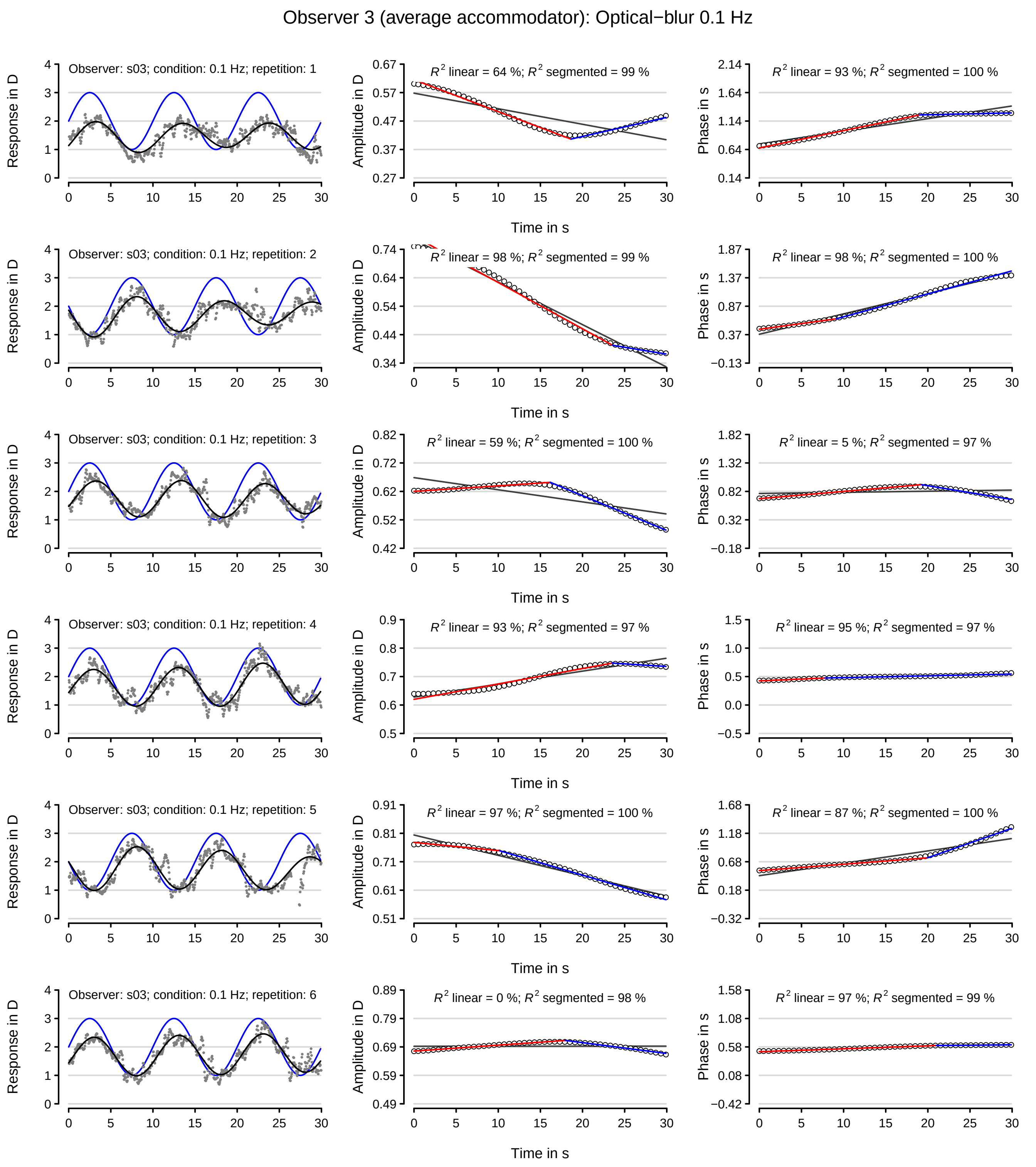

Observer 3 (average accommodator): Optical−blur 0.1 Hz
Observer: s03; condition: 0.1 Hz; repetition: 1
R^2 linear = 64 %; R^2 segmented = 99 %
R^2 linear = 93 %; R^2 segmented = 100 %
Observer: s03; condition: 0.1 Hz; repetition: 2
R^2 linear = 98 %; R^2 segmented = 99 %
R^2 linear = 98 %; R^2 segmented = 100 %
Observer: s03; condition: 0.1 Hz; repetition: 3
R^2 linear = 59 %; R^2 segmented = 100 %
R^2 linear = 5 %; R^2 segmented = 97 %
Observer: s03; condition: 0.1 Hz; repetition: 4
R^2 linear = 93 %; R^2 segmented = 97 %
R^2 linear = 95 %; R^2 segmented = 97 %
Observer: s03; condition: 0.1 Hz; repetition: 5
R^2 linear = 97 %; R^2 segmented = 100 %
R^2 linear = 87 %; R^2 segmented = 100 %
Observer: s03; condition: 0.1 Hz; repetition: 6
R^2 linear = 0 %; R^2 segmented = 98 %
R^2 linear = 97 %; R^2 segmented = 99 %
Response in D
Amplitude in D
Phase in s
Time in s

Observer 3 (average accommodator): Optical−blur 0.2 Hz

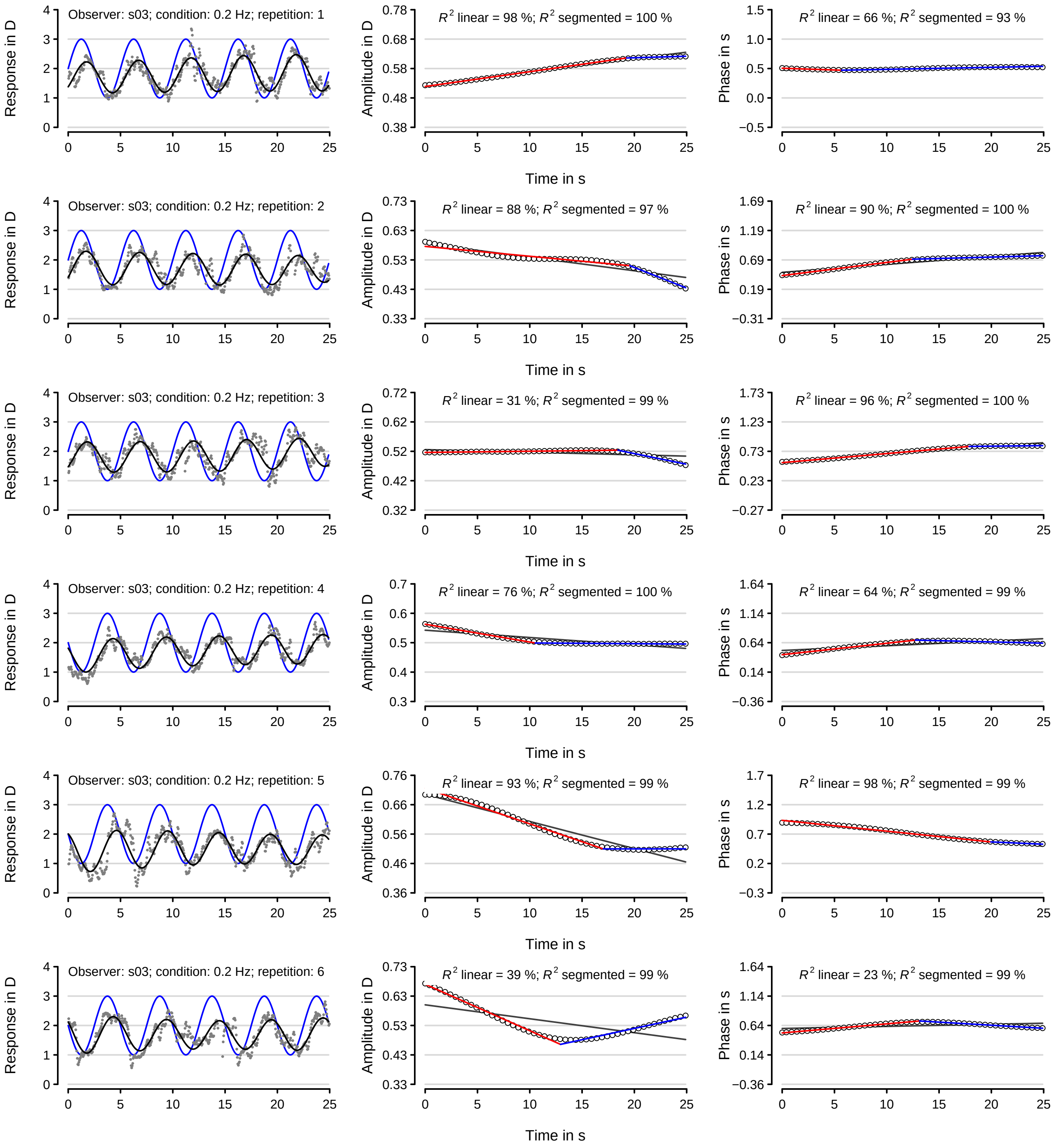

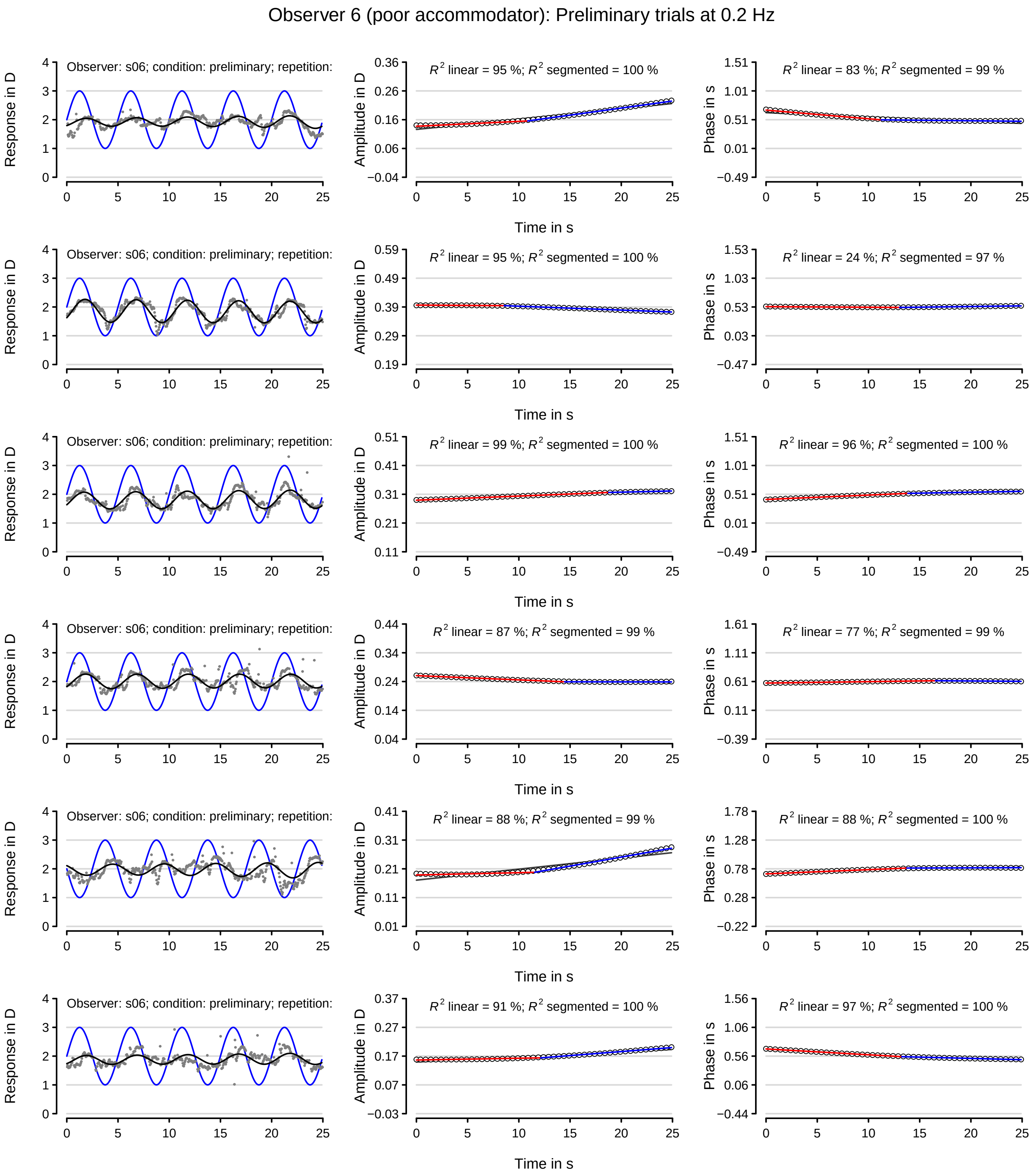
Observer 6 (poor accommodator): Preliminary trials at 0.2 Hz
Observer: s06; condition: preliminary; repetition:
Response in D
Amplitude in D
Phase in s
Time in s
R^2 linear = 95 %; R^2 segmented = 100 %
R^2 linear = 83 %; R^2 segmented = 99 %
R^2 linear = 95 %; R^2 segmented = 100 %
R^2 linear = 24 %; R^2 segmented = 97 %
R^2 linear = 99 %; R^2 segmented = 100 %
R^2 linear = 96 %; R^2 segmented = 100 %
R^2 linear = 87 %; R^2 segmented = 99 %
R^2 linear = 77 %; R^2 segmented = 99 %
R^2 linear = 88 %; R^2 segmented = 99 %
R^2 linear = 88 %; R^2 segmented = 100 %
R^2 linear = 91 %; R^2 segmented = 100 %
R^2 linear = 97 %; R^2 segmented = 100 %

Observer 6 (poor accommodator): Optical−blur 0.05 Hz

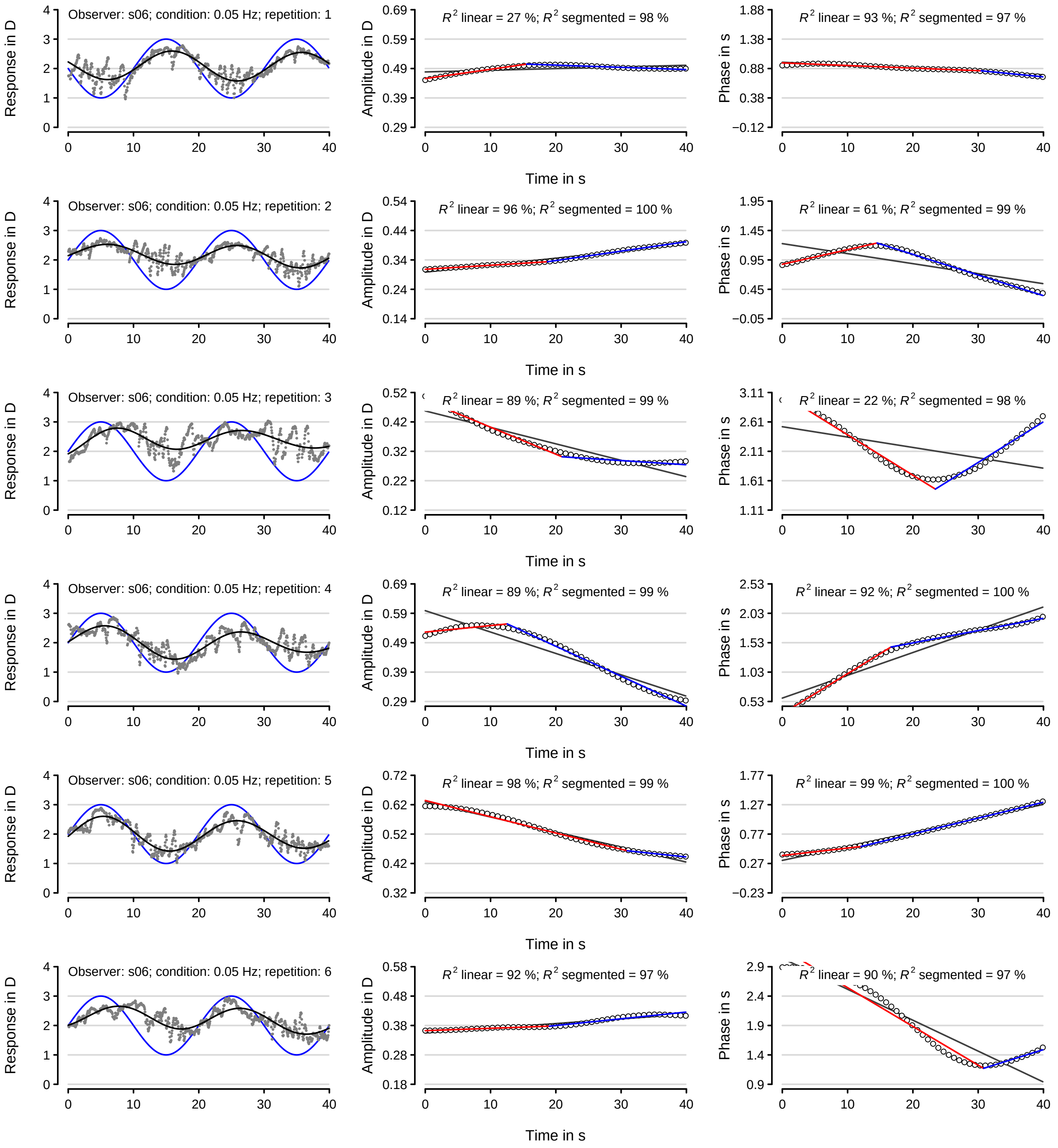

Observer 6 (poor accommodator): Optical−blur 0.1 Hz

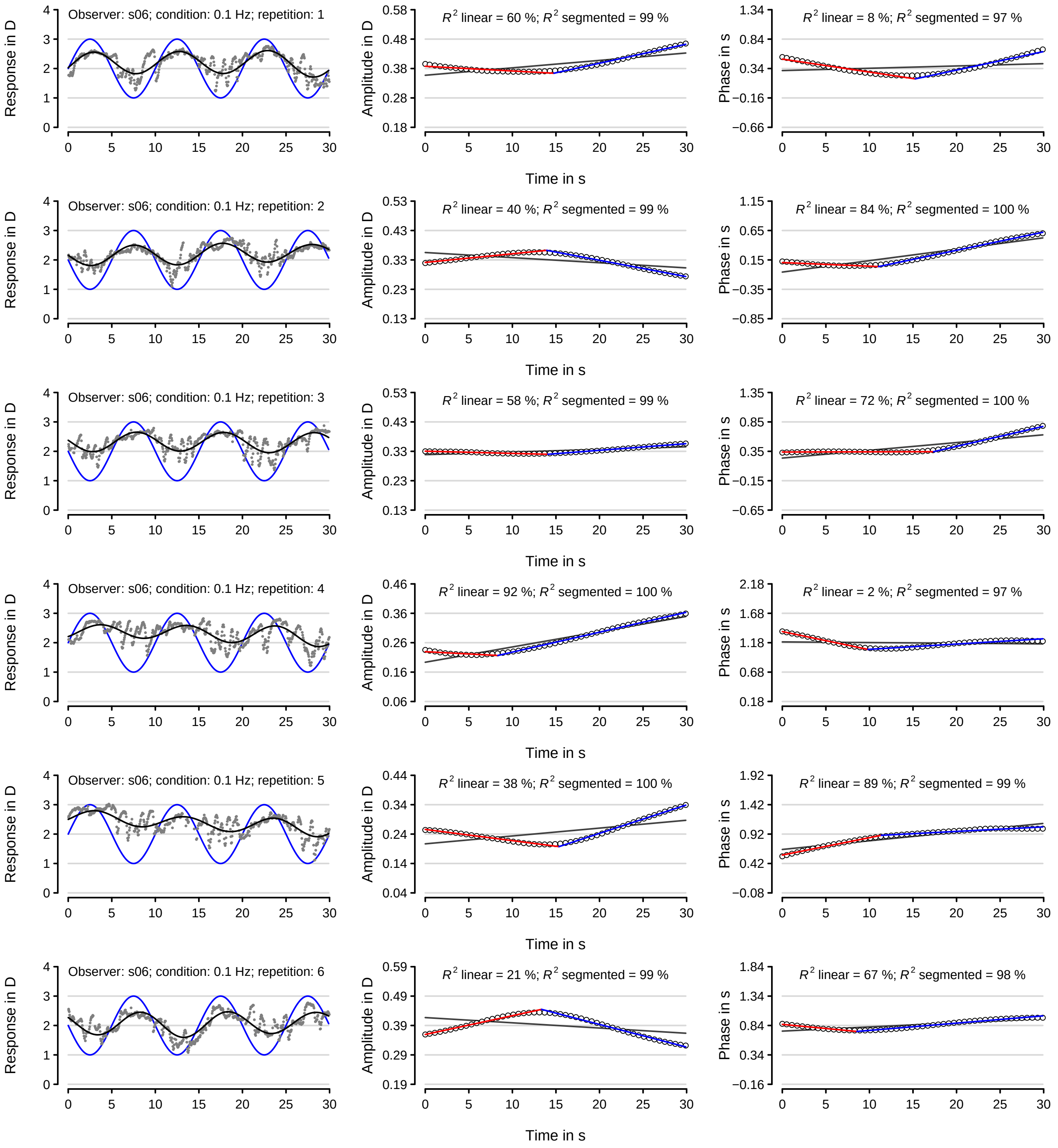

Observer 6 (poor accommodator): Optical−blur 0.2 Hz

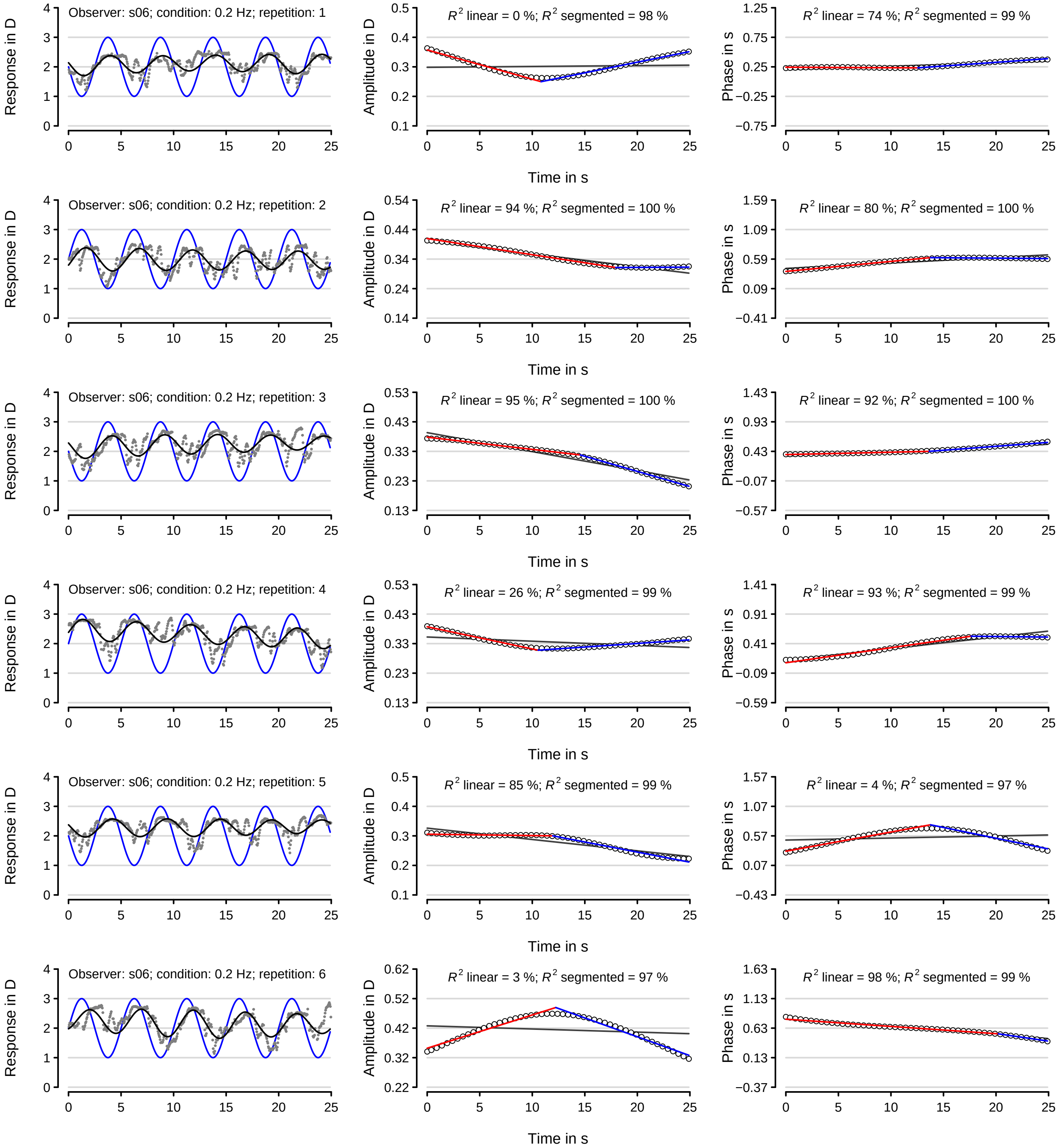

Supplement: Supplementary file 1 — Supplementary Information. [file 41598_2021_94642_MOESM1_ESM.pdf]
